# Syphilis and pregnancy

**DOI:** 10.61622/rbgo/2024FPS09

**Published:** 2024-09-23

**Authors:** Geraldo Duarte, Patrícia Pereira dos Santos Melli, Angélica Espinosa Miranda, Helaine Maria Besteti Pires Mayer Milanez, Maria Luiza Menezes, Ana Gabriela Travassos, Regis Kreitchmann

**Affiliations:** Faculdade de Medicina de Ribeirão Preto Universidade de São Paulo Ribeirão Preto SP Brazil Faculdade de Medicina de Ribeirão Preto, Universidade de São Paulo, Ribeirão Preto, SP, Brazil.; Hospital das Clínicas Faculdade de Medicina de Ribeirão Preto Universidade de São Paulo Ribeirão Preto SP Brazil Hospital das Clínicas, Faculdade de Medicina de Ribeirão Preto, Universidade de São Paulo, Ribeirão Preto, SP, Brazil.; Faculdade de Medicina Universidade Federal do Espírito Santo Vitória ES Brazil Faculdade de Medicina, Universidade Federal do Espírito Santo, Vitória, ES, Brazil.; Faculdade de Ciências Médicas Universidade Estadual de Campinas Campinas SP Brazil Faculdade de Ciências Médicas, Universidade Estadual de Campinas, Campinas, SP, Brazil.; Faculdade de Ciências Médicas Universidade de Pernambuco Recife PE Brazil Faculdade de Ciências Médicas, Universidade de Pernambuco, Recife, PE, Brazil.; Faculdade de Medicina Universidade do Estado da Bahia Salvador BA Brazil Faculdade de Medicina, Universidade do Estado da Bahia, Salvador, BA, Brazil.; Universidade Federal de Ciências da Saúde de Porto Alegre Porto Alegre RS Brazil Universidade Federal de Ciências da Saúde de Porto Alegre, Porto Alegre, RS, Brazil.

## Abstract

•Although congenital syphilis has a known etiological agent, accessible diagnosis and low-cost, effective treatment with low fetal toxicity, it continues to challenge obstetric and antenatal care services.

•The increasing rates of syphilis in the general population have direct repercussions on the increase in cases of congenital syphilis, a situation of objective interest for public health.

•Although transforming the recording of syphilis and congenital syphilis into notifiable diseases improved the records and has made it possible to measure the occurrence of these diseases and create solutions, no effects on reducing their frequency have been reached yet.

•The failure to control syphilis/congenital syphilis is multifactorial, and associates variables that range from the deficiency in teaching about these diseases in schools and in the training system of the various health professional segments, as well as the lack of rigid policies for quality control from antenatal care until the clinical follow-up of children exposed to *Treponema pallidum* during pregnancy.

•To date, benzathine penicillin is the only antimicrobial accepted as effective by the main health authorities on the planet for the treatment of syphilis in pregnant women.

•The fear of anaphylaxis in response to the treatment of syphilis with benzathine penicillin is an important factor hindering the prompt and correct treatment of pregnant women with syphilis, even though health authorities have made efforts to face the problem with solid arguments, still insufficient to resolve the question.

•Although specific protocols are published, the failure to control the treatment of syphilis in pregnant women is still observed with high frequency, indicating and reinforcing a failure in the quality control of these care principles.

The National Specialized Commission on Infectious Diseases of the Brazilian Federation of Gynecology and Obstetrics Associations (Febrasgo) endorses this document. Content production is based on scientific evidence on the proposed topic and the results presented contribute to clinical practice.

## Recommendations

- Establish policies encouraging group meetings for pregnant women that include syphilis among topics for discussion and emphasize prophylactic measures to prevent it.
- Remember that most cases of syphilis are diagnosed in the latent phases (recent and late). Therefore, the initiative of the antenatal professional in the serological search for diagnosis is crucial.
- As the diagnosis of syphilis is biphasic, give preference to treponemal tests for screening.
- Before starting treatment for syphilis in pregnant women, it is necessary to establish the evolutionary phase of syphilis, whether recent or late, as the dose of benzathine penicillin and duration of treatment are different.
- For the treatment of syphilis in the recent phase (primary, secondary or recent latent), a single dose of benzathine penicillin 2,400,000 IU intramuscularly is used.
- For the treatment of syphilis in the late phase, benzathine penicillin 2,400,000 IU intramuscularly is used weekly for three sequential weeks.
- Establish local (hospital) care protocols for the correct diagnosis of penicillin allergy and desensitization in confirmed cases. Give preference to the coordination of this service by allergy specialists. If this is not possible in the hospital where you work, check the referral flow for pregnant women who need this resource.
- Syphilis treatment control must be performed with nontreponemal tests, and a decline of two dilutions is expected in three months, three dilutions in six months and negative or stable in low dilutions (two sequential monthly tests).
- Establish the best strategy in each service to include the partner in antenatal care.

## Background

Syphilis is an infectious disease caused by the *Treponema pallidum*. Its prevalence has challenged science over time, since it has a defined etiological agent, accessible diagnosis, effective and low-cost treatment and is sensitive to common hygienic measures.^(1)^ It presents systemic evolution from the initial phase, after an extremely variable incubation period that depends on the host’s innate defenses and immune response.^(2,3)^ It can evolve chronically with discreet or exuberant clinical manifestations alternating with clinically silent periods called the latent phase.^(4)^ Although *Treponema pallidum* infection through blood transfusion or contaminated piercing material is feasible, the main and most important routes of transmission are sexual (genital, oral and anal) and vertical, which can result in fetal death or congenital syphilis.^(5)^ This fact contributes to making syphilis in pregnant women a global public health problem.^(6,7)^ Its control is one of the objectives of the World Health Organization (WHO) and also included in the sustainable health agenda of the Pan American Health Organization (PAHO).^(8-10)^

Even though they are considered from the perspective of the same etiological agent, the approach to acquired syphilis is very different from the approach to congenital syphilis. There are marked differences in the pathophysiological process, the organic and immune response of patients, clinical manifestations, diagnosis and treatment. Congenital syphilis is based on the vertical transmission of the etiological agent, and the scope of this text will be to record all the strategies and resources that obstetricians can access to prevent it. Nowadays, congenital syphilis is considered a clear inverse marker of the quality of antenatal care, i.e., the higher the occurrence rate the more inadequate the quality of antenatal care offered.^(11)^

### How important is it to know the biomolecular details of *Treponema pallidum*?

*The Treponema pallidum (subspecies pallidum), a spirochete-shaped bacterium, was described in 1905*.^(12)^ It does not stain using the Gram technique and does not grow in artificial culture media. It has a double phospholipid membrane and most of the lipid components are present on the inner surface of the membrane.^(3)^ The final result of the genomic sequencing of *Treponema pallidum* was released in 1998,^(13)^ which provided support to understand the resistance profile of this bacterium to antimicrobials and^(14)^ determine strains responsible for epidemic outbreaks^(15)^, and for vaccine studies.^(16)^

### How has the frequency of syphilis varied in the population and in pregnant women?

**Figure 1:**
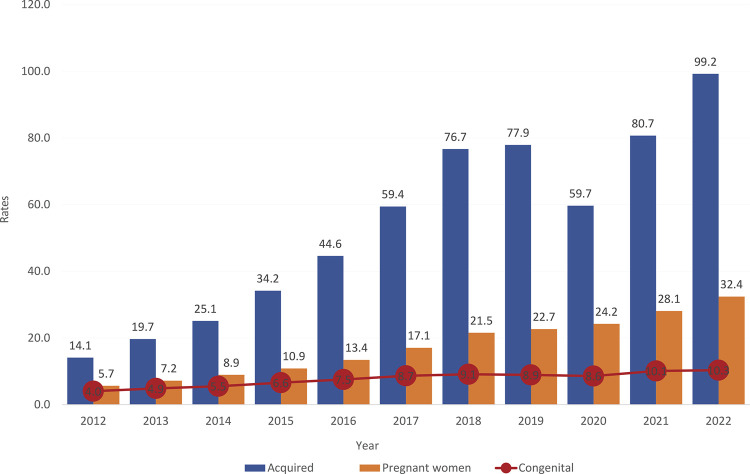
Acquired syphilis detection rate (per 100,000 inhabitants), syphilis detection rate in pregnant women and congenital syphilis incidence rate (per 1,000 live births)

Source: Epidemiological Bulletin – Syphilis (2023).^(21)^

Syphilis is widely distributed disease across the planet and no nation is free from its occurrence.^(17)^ The resurgence of syphilis has been associated with numerous social and behavioral variables, including greater sexual freedom, sexual tourism and contraceptive security. According to an estimate by the WHO (2021),^(18)^ 7.1 million new cases of syphilis occur in the world each year and approximately 90% of these in developing countries.^(19)^ However, this is also a problem in industrialized countries.^(20)^ Considering the occurrence of syphilis in the Brazilian population (in adults, pregnant women and congenital syphilis) in the year 2021, 167,523 cases of acquired syphilis were registered in adults (78.5 per 100,000 inhabitants); 74,095 cases of syphilis in pregnant women (27.1% live births); 27,019 cases of congenital syphilis (9.9% live births) and 192 deaths due to congenital syphilis (0.07% live births).^(21)^ The annual evolution of the prevalence of these parameters can be assessed in figure 1.

The notification of syphilis and congenital syphilis complies with current regulations that define the National List and Compulsory Notification of Diseases, Conditions and Public Health Events in public and private health services throughout the national territory and is based on Consolidation Ordinance number 204 of February 17, 2016.^(22)^

### How important is it to know the pathophysiology and clinical picture of syphilis in its various phases?

The entry of *Treponema pallidum* into the body determines the location where the first syphilis lesion will develop. It has a variable incubation period (two to 10 weeks), but the primary lesion of syphilis, known as protosyphiloma or hard chancre, develops in three to four weeks, on average.^(23)^ It is characterized by being a shallow painless ulcer with a clean bottom, hard edges and an erythematous base.^(3,24)^ Most of the time it manifests as a single lesion, but it can present multiple lesions, including lesions outside the genital area (mouth and anus, more frequently).^(2,25)^ The clinical diagnosis of syphilis at this stage is more frequent in men, in whom the genital lesion is visible, which may not occur in women when the primary lesion is located in the vagina or uterine cervix.^(26)^ In this case, the lesion can be visualized only with an examination using a gynecological speculum. When syphilis is treated at this stage, the lesion usually disappears in less than a week. However, without treatment, the natural evolution of this lesion is its disappearance without scars in an extremely variable time, from two to three weeks on average, when it affects immunologically healthy people. However, it may last up to eight weeks in people with some degree of immune impairment. Approximately 10 days after the appearance of protosyphiloma, in approximately half of the cases, painless satellite adenomegaly is observed without inflammatory signs and non-fistulizing.^(27)^ At this stage of the disease, the immune system normally does not yet exhibit a response that can be safely measured by serological tests, as only 15% of serological tests are positive. However, the biggest problem is that it may be serological memory resulting from prior episode(s) of syphilis.

Considering the natural evolution of untreated syphilis, in the absolute majority of cases, secondary lesions appear after the primary lesion disappears and starts the immune response against its etiological agent. Clinical manifestations arise as a result of the hematogenous dissemination of *Treponema pallidum* on a large scale,^(27)^ justifying the highest rate of vertical transmission of this microorganism observed at this stage. The moment when secondary lesions appear depends basically on two factors: the virulence of the treponema and the host’s systemic response to the local accumulation of treponema in susceptible tissues. For these reasons, the time for the onset of clinical manifestations of secondary syphilis is extremely variable.^(25)^ However, on average, it ranges between six and 12 weeks after the infectious event, with an average of seven to eight weeks. In the integumentary system it presents as multiform and varied lesions such as rash, papules and roseolas and rarely pustular (syphilides). It may also present with madarosis, patch alopecia and paronychia.^(2,24)^ In regions of friction or very humid areas, lesions called condyloma lata of syphilis may appear, which are relatively common in the periorificial regions (genitals, anus and mouth). Secondary syphilis lesions are rich in *Treponema pallidum*. The more common systemic manifestations of secondary syphilis, are: asthenia, anorexia, fever, generalized micropolyadenopathy, myalgia, arthralgia, headache, meningitis, meningovascular syphilis, iridocyclitis, hepatitis, splenomegaly, periostitis and glomerulonephritis, among others.^(2,3)^

•With or without treatment, the secondary clinical manifestations of syphilis disappear within a year of evolution, establishing a long clinically silent period, with rare cases of recrudescence in the first year of latency. This asymptomatic period of syphilis is also characterized by the gap in scientific knowledge it represents, questioning what causes the clinical signs of secondary syphilis to be controlled without avoiding the late onset of serious lesions at the tertiary phase of the disease.
•Tertiary syphilis begins with the first clinical manifestation of this phase of the disease. Such a broad involvement results in a vast list of clinical changes dominated by integumentary, visceral, bone and nervous system changes. Integumentary changes are dominated by gumma lesions; visceral by ocular and cardiovascular changes (ostial insufficiency and aneurysm); and bone by destructive lesions of its tissue.^(2)^ The involvement of the central nervous system (CNS) in tertiary syphilis occurs due to an inflammatory reaction, affecting vessels, meninges and the parenchyma. The involvement is variable, causing neurological and psychiatric signs and symptoms, such as headache, insomnia, anxiety, obtundation, hallucination, dementia, paresis and tabes dorsalis.^(28)^

### How is syphilis classified temporally and clinically?

Acquired syphilis is classified according to its temporal evolution into recent syphilis and late syphilis, divided by a period of latency (absence of clinical manifestations). Therefore, it is characterized by clinical phases or stages^(4,27,29)^ as follows:

**•Recent syphilis:** Recent syphilis lasts up to one year after the infectious event. It is characterized by two phases: one symptomatic (primary and secondary lesions of syphilis) and the other asymptomatic (recent latent syphilis). In the symptomatic phase, the infectious lesions are transient and rich in *Treponema pallidum*, and disappear within one year of the disease’s evolution. The first lesion that appears is hard chancre, which settles at the site of *Treponema pallidum* inoculation.^(5)^ The average incubation period is three to four weeks. Since this is the first lesion of the disease, it is also called primary lesion of the recent syphilis (also called primary syphilis). On average, this lesion disappears within one to three weeks in healthy people, even without treatment.

In general, around three to five weeks after the onset of the primary lesion of recent syphilis, secondary syphilitic lesions appear (also called secondary syphilis), characterized by multiform lesions in their clinical presentation, which disappear within a year of disease evolution counting from the infectious event.

In the period between the disappearance of the secondary lesions of recent syphilis and the completion of one year of evolution of the infection, begins what is known as recent latent syphilis, characterized by the absence of clinical manifestations. Serological tests are positive in secondary syphilis.

**•Late syphilis:** Late syphilis begins one year after the infectious event and has an extremely variable duration, depending on the interaction and the patient’s immune response. According to some authors, it can last up to more than 40 years.^(23,27)^ It is divided into late latent syphilis (clinically silent) and late syphilis itself, after the onset of late lesions of the disease (gumma, bone, cardiovascular, neurological lesions, among others). Late latent syphilis begins one year into the disease, at the end of the recent latent phase. It is the longest duration of syphilis, with records of more than 30 years. It ends with the onset of the first clinical manifestation of the late phase of syphilis. In general, serological tests remain positive at this stage of the disease. Figure 2 represents a practical synopsis of the clinical and temporal classification of syphilis.
  **Figure 2:**
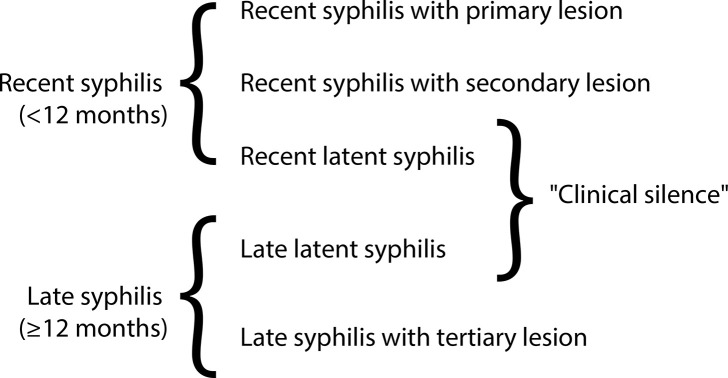
Synopsis of the classification of clinical and temporal phases of syphilis

Source: Prepared by the author

### How is the clinical diagnosis made?

In the clinical diagnosis of acquired syphilis, it is important to remember the value of anamnesis and to know the pathophysiology of the disease, its clinical and evolutionary classification, in addition to the confirmed history of possible treatments in the past.^(1)^ Recent syphilis is characterized by lesions rich in *Treponema pallidum*, with rare exceptions. The first lesion that appears is hard chancre and its characteristics have already been described previously. Next, considering the natural history of the disease, the more frequent secondary manifestations are integumentary lesions such as roseola, maculopapular erythema, condyloma lata, palmar and plantar erythematodesquamative lesions, madarosis, clearing alopecia and nail changes. Systemic manifestations are less frequent and represent organic responses to *Treponema pallidum* aggression. Considering the low rate of occurrence of these changes, the most cited are: asthenia, anorexia, fever, generalized micropolyadenopathy, myalgia, arthralgia, headache, meningismus, iridocyclitis, hepatitis, splenomegaly, periostitis and glomerulonephritis.^(2,24,27)^ Note that neurosyphilis is not a characteristic of tertiary syphilis, and may also appear in the set of lesions of secondary syphilis in percentages that do not exceed 1%.^(25)^

After a variable period of late latency that can last decades, tertiary lesions of syphilis appear, i.e., inflammatory lesions of varying severity depending on the location where they appear. In the integumentary system, destructive, circumscribed and non-infectious nodular-ulcerative lesions, called gummas, are observed. They can be single, multiple and of varying dimensions, and can also affect the bones. In the CNS, clinical conditions vary from asymptomatic to more severe cases of dementia, paresis, paresthesia, headache, symptoms of meningeal involvement, involvement of the cranial nerves and even tabes dorsalis.^(30)^ The organic changes of late syphilis on the cardiovascular system also fundamentally result from changes in inflammatory phenomena in the aorta, which may be asymptomatic, but also have the potential to cause chest pain, aortic regurgitation, heart failure, coronary ostial stenosis, angina and aneurysm.^(27,28)^

### How is laboratory diagnosis made?

The laboratory diagnosis of syphilis both to confirm a diagnostic hypothesis and to search for people in the latent phase of the disease is essential, especially in pregnant women. Based on the evolutionary characteristics of syphilis, the method to be used for the laboratory diagnosis will be indicated depending on the clinical stage of the patient. Therefore, clinical manifestations will provide support for choosing the best option, the one that determines the best performance of the laboratory resource used. The tests are divided into two broad categories: direct tests, which directly investigate the presence of *Treponema pallidum* or its DNA; and tests that investigate the immune response against the microorganism through serum tests.^(27,29)^

Direct examinations include the search for *Treponema pallidum* in samples collected directly from the lesions, and are indicated when hard chancre (primary lesion of syphilis) is suspected. Direct examinations may be influenced by the experience of the observer, the quantity of treponemas in the material collected, and by the presence of secondary bacterial infections.^(31)^ From a practical point of view, none of the direct observation tests for the microorganism are indicated in oral and/or rectal lesions given the presence of non-pathogenic spirochetes that may potentially falsify the diagnosis. Among these techniques, dark-field microscopy (performed at the time of obtaining the sample, observing the live and mobile microorganism) and techniques that use dyes (silver impregnation or direct immunofluorescence) can be used.^(26,27)^ The identification of *Treponema pallidum* DNA using molecular biology techniques (polymerase chain reaction – PCR),^(32)^ is an accessible possibility in large private laboratories in the country, but not yet available to patients users of the Brazilian Unified Health System (SUS). It can also be used for diagnosis in oral and anal lesions.^(26)^

Considering the serological diagnosis of syphilis, there are two large groups of tests: treponemal tests (TTs) and nontreponemal tests (NTTs). They are characterized by the search for antibodies against *Treponema pallidum* in samples of whole blood, serum, plasma or liquor.^(31)^ Remember that the serological diagnosis of syphilis is biphasic and requires confirmation of the initial test.^(26)^

Treponemal tests detect specific antibodies produced against *Treponema pallidum* antigens. They become positive, on average, one to three weeks after the appearance of the hard chancre. They are excellent diagnostic methods, although not suitable for controlling treatment. Currently, the most used are the Fluorescent Treponemal Antibody Absorption (FTA-Abs), the Treponema pallidum Haemagglutination test (TPHA) and the Enzyme-Linked Immunosorbent Assay (Elisa) with its variants, including chemiluminescence.^(31)^ Rapid tests, characterized as a powerful diagnostic resource for syphilis, are also TTs, and normally developed on an immunochromatography platform.^(29,33)^

Nontreponemal tests use cardiolipin as a reacting antigen, demonstrating the formation of anti-lipid antibodies. They become positive, on average, two to three weeks after the appearance of the hard chancre. They are flocculation tests and can be used for both diagnosis and therapeutic follow-up, since their results are qualitative and expressed quantitatively by serial titrations. Among a multitude of possibilities, the most used tests are the Venereal Disease Research Laboratory (VDRL), the Rapid Plasma Reagin (RPR) and the Toluidine Red Unheated Serum Test (TRUST). They are used for both serum and cerebrospinal fluid analysis.^(29,31)^

### What are the differential diagnoses?

The polymorphism of the clinical manifestations of syphilis, the possibility of involvement of practically all organs of the body and the temporal variations of these clinical events alert to the large number of differential diagnoses of this disease.^(24,27,34)^ In recent syphilis, hard chancre must be differentiated from all other diseases that cause erosion and/or ulceration in the genitalia, each with its own clinical variation, which can provide guidance on the laboratory test that should eventually be used to confirm the clinical hypothesis of the diagnosis suggested. As an example, the erosions observed in cases of chancroid, herpes type 2, Behçet’s syndrome, pharmacodermias, among others, have an evident painful component supporting other diagnoses.^(1)^ Considering the secondary lesions of recent syphilis, syphilitic roseola and papular syphilides must be differentiated among the numerous diseases that cause rashes, such as pityriasis rosea, pharmacodermias, viruses, leprosy, collagenosis, urticaria, among others.^(30,34)^ The manifestations of late syphilis also allow numerous differential diagnoses; for example, gumma lesions must rule out tuberculosis, leishmaniasis, sporotrichosis, among other granulomatous diseases. Given its special characteristics, neurosyphilis allows for several differential diagnoses, such as: congenital aneurysm, tuberculous meningitis, intracranial tumor and psychiatric and emotional disorders.^(28)^ In these cases, laboratory diagnosis is crucial to establish the diagnosis and justify the treatment.

### Is there an influence of the clinical stage of syphilis on vertical transmission rates?

Regarding the epidemiology of congenital syphilis, note that the rate of vertical transmission of *Treponema pallidum* varies according to the duration of the disease and the moment of pregnancy when the infectious event occurred. Recent syphilis (primary and secondary syphilis) is known to offer a greater probability of vertical transmission of *Treponema pallidum*, reaching up to 80%.^(35)^ This is justified by the significantly higher presence of microorganism in maternal blood circulation at this stage of the disease. However, latent syphilis (early and late) also presents risks of transplacental transmission, although at lower rates than in the recent phase of the disease with clinical manifestations. Considering all these particularities and all phases of syphilis, it is accepted that 62-80% of cases of untreated maternal syphilis may present some type of compromised gestational prognosis represented by miscarriage and fetal death (21% to 26%), neonatal death (6% to 11%), congenital syphilis (9% to 25%) and prematurity/growth restriction (11% to 18%).^(36-38)^

### How and why should pregnant women be screened for syphilis?

**Figure 3:**
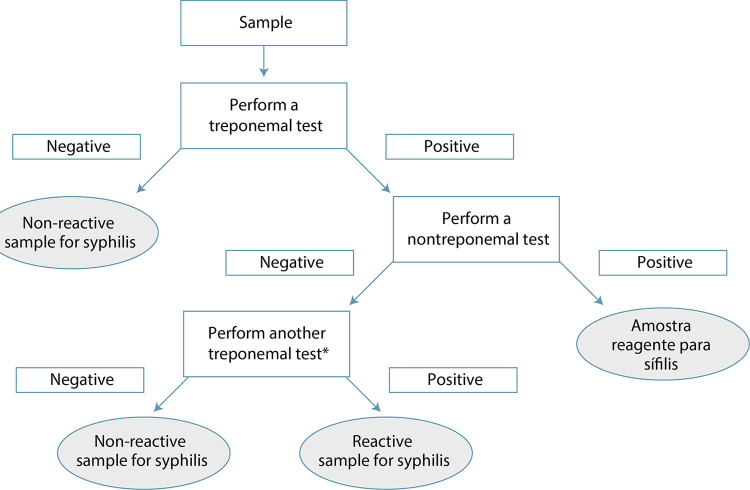
Inverted flowchart for syphilis screening in pregnant women starting with the treponemal test

Considering the diagnosis of syphilis in pregnant women, the vast majority of cases appears to be diagnosed in the latent phase, without clinical manifestations. With this information and without screening, it will not be possible to prevent congenital syphilis. Diagnosis at this stage is based on the reactivity of serological tests within a clinical and epidemiological context to conclude a definitive diagnosis. Only the combination of all this information will allow the correct diagnostic assessment of each case and then the indication of appropriate treatment, avoiding the perverse effects of syphilis on the prognosis of pregnancy.^(1,39)^ There are two strategies for diagnostic screening depending on the sequence of serological tests that will be used, remembering that this is two-phase diagnosis; screening followed by confirmation. Considering the sensitivity of the tests, it is recommended to start the investigation with TT (also called reverse flow), including rapid tests here. If the tested sample is not reactive, this result is accepted to rule out the sample’s seropositivity for syphilis at that time.^(31)^ In any case, this screening must be repeated around week 28 of pregnancy and at birth. If the sample is reactive, it must be confirmed by an NTT. In cases of agreement on the reactivity of the two tests, in light of clinical and epidemiological information, the diagnosis of syphilis for this pregnant woman is considered as confirmed. If the first test of the sample is reactive and the second test is non-reactive, a third test is necessary, in this case a TT with a different principle from that of the previously used TT. This third result will be considered definitive, thus determining the serological diagnosis of the case (Figure 3). Obviously, any result must consider the pregnant woman’s documented information about any previous treatments, which allow these samples to be positive.^(39)^

*Treponemal test based on a different technology from that of the previous test. The rapid test can be used.

Source: Ministry of Health (2021).^(31)^

**Figure 4:**
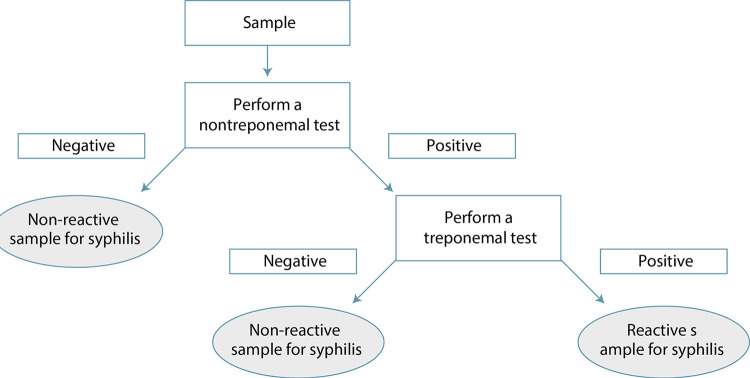
Flowchart for syphilis screening in pregnant women starting with a nontreponemal test

Source: Ministry of Health (2016).^(31)^

If the choice is to start screening with an NTT (classic flow), non-reactive samples end the diagnostic research for that moment in pregnancy.^(31)^ As previously stated, in this screening flowchart in pregnant women with negative samples, screening should be repeated around week 28 of pregnancy and at birth.^(39)^ A reactive result needs to be confirmed by a TT, and such a result, in light of clinical and epidemiological information, will be considered for the diagnosis of the case (Figure 4). Over time, the humoral defense against syphilis may decline and the cellular defense becomes progressively more prominent in late syphilis. This information is important, since these are pregnant women, and it indicates that low antibody titers do not rule out active syphilis.^(27)^

Pregnant women should be tested for syphilis at least in the first antenatal visit, at the beginning of the third trimester and at the time of admission for delivery. Women who experience miscarriage, fetal loss or those subjected to situations of risk exposure or sexual violence must also be tested.^(1,39)^ Pregnant women who live in difficult-to-reach communities and have only undergone a positive test for syphilis, given the limitation, should be considered for treatment. Until proven otherwise and in the absence of appropriate and documented treatment, these pregnant women must be treated at the time of antenatal consultation, delivery or in the event of an abortion. In these situations, it is not prudent to wait for confirmatory tests. Delaying treatment due to waiting for the result of a complementary test causes the professional to waste time and opportunities to avoid cases of congenital syphilis.^(39)^ Syphilis screening among pregnant women is considered one of the most cost-effective health strategies of the antenatal period, especially for developing countries.^(40,41)^ This screening strategy allows the early treatment, as well as avoiding miscarriage, fetal death, prematurity, intrauterine growth restriction and congenital syphilis.^(42)^

### How is syphilis treated in pregnant women?

The recommended treatment for syphilis in pregnant women is benzathine benzylpenicillin, a highly effective medication against *Treponema pallidum*, whose percentage of sensitivity to penicillin remains unquestioned in practice in Brazil. The slow release of benzathine penicillin coincides with the slow replication rate of the microorganism, variables that reinforce the important role of this medication in the treatment of syphilis. It is also a safe option for use in pregnant women, with no documentation of harmful effects on the embryo or fetus (Food and Drug Administration category B). Other medications can be used to treat syphilis outside of pregnancy (tetracycline, macrolides and ceftriaxone). However, for pregnant women, the only globally accepted option to correctly treat the mother and fetus continues to be benzathine penicillin used up to 30 days before birth at the dose indicated for the stage of the disease.^(43,44)^ The doses of benzathine penicillin vary according to the stage of the disease. In recent syphilis, up to one year after the infectious event (primary/secondary lesions and recent latent syphilis), the intramuscular single dose of 2,400,000 IU is used.^(39)^ Some services recommend an additional dose one week after the first dose.^(4,45)^ For the treatment of late syphilis, one year after the infectious event (late latent phase and tertiary syphilis lesions), the dose is 2,400,000 IU administered weekly, for three consecutive weeks. The interval between doses should not exceed nine days. If this occurs, the regimen must be restarted.^(46)^ In the case of suspected neurosyphilis, it is important to remember that benzathine penicillin does not cross the blood-brain barrier, and priority should be given to the use of intravenous crystalline penicillin (4,000,000 IU intravenously every four hours or in continuous infusion totaling 24,000,000 IU/day) for 10-14 days in the hospital.^(29)^ In these cases, joint follow-up with an infectious disease specialist or neurologist is important.

Objectively considering the situation of shortage of benzathine penicillin, the use of procaine penicillin or ceftriaxone is recommended for the treatment of pregnant women with syphilis. Procaine penicillin can be used at a dose of 600,000 IU/day intramuscularly for 10-14 days (recent or late syphilis, respectively). For ceftriaxone, the dose is 1 g/day intramuscularly, also for 10-14 days (recent or late syphilis, respectively). Studies carried out with ceftriaxone for the treatment of syphilis in pregnant women still do not provide complete safety for its use with a view to indisputable control of congenital syphilis, which is why it is not the recommended medication for the treatment of syphilis in pregnant women.^(4,44)^ In cases of treatment with ceftriaxone, it is recommended to investigate the newborn as a potential case of congenital syphilis. In turn, macrolides/azalides (erythromycin/azithromycin) do not cross the placental barrier in adequate concentrations, in addition to consistent reports in the literature of history of resistance of *Treponema pallidum* to these drugs.^(47,48)^

### What are the procedures when a pregnant woman with syphilis is allergic to penicillin?

It seems to be consensual that the fear of adverse reactions cannot be an impediment to the administration of benzathine penicillin in health services, especially in Primary Care.^(49)^ With this concern, the Brazilian Ministry of Health has objectively invested in the training of health teams, preparing them to care for patients who eventually present some complication.^(29,50)^ Without scientific information, there is no way to face the fear of allergy to penicillin, one of the important factors in limiting the correct treatment of syphilis in pregnant women. The first step is to inform about the population risk of these complications and inform the patient that serious complications are rare. During regular medical history taking, penicillin allergy is reported by 3%-5% of pregnant women, but it is important to objectively check the “allergy” history, as the patient may incorrectly assume that a non-allergic side effect (e.g. example, nausea or vomiting) is of allergic origin. The main symptoms of concern are (immediate) immunoglobulin E (IgE)-mediated responses such as urticaria, angioedema or anaphylaxis with airway obstruction, bronchospasm or hypotension. Anaphylaxis, considered a serious event, has a low frequency and does not exceed 0.002% (95% confidence interval [CI]: 0%-0.003%) of cases.^(51)^ Remember that anaphylactic events can also occur at a much higher frequency due to several triggering factors, including foods (nuts, seafood, food coloring) and medications more commonly used than penicillin, such as anti-inflammatories, anesthetics, neuromuscular blockers and sulfa drugs.^(52)^ Most cases grossly identified as suspected of being allergic to penicillin lack careful anamnesis to qualify this change. In view of this, an objective and specific anamnesis is fundamental and imperative to adequately obtain this information. This decision was based on the high number of suspected cases of allergy to penicillin sent for desensitization, noting that almost all of them were ruled out solely by anamnesis.^(53)^ After confirming that the clinical history is indeed suggestive of allergy to penicillin, the pregnant woman should be referred to reference services. The current protocols aim to better characterize allergic processes and can rule out allergy to penicillin in the absolute majority of the most suspected cases.^(54)^ If the allergy is confirmed, the best option is desensitization and application of the first dose of penicillin.^(55,56)^ It is strongly recommended to perform desensitization in a hospital setting with allergy specialists. According to the Australasian Society of Clinical Immunology and Allergy (2020)^(57)^ and the Resuscitation Council UK (2021),^(58)^ if the anaphylactic reaction occurs in a patient with no previous history of allergy to penicillin, adrenaline is the drug of choice for immediate treatment and the first ventilatory support measures must be quickly instituted.

### What is the Jarisch-Herxheimer reaction?

The Jarisch-Herxheimer reaction is an organic response to the treatment of various infections. Although this is the initial and more frequent description after the treatment of syphilis, it is also associated with the treatment of other diseases caused by spirochetes (Lyme disease, leptospirosis),^(59)^ fungi and protozoa.^(60)^ The common factor of the Jarisch-Herxheimer reaction is the onset triggered by the use of antimicrobials, most notably penicillin, but it can also be triggered after the use of other antibacterials such as tetracyclines, cephalosporins, quinolones, carbapenems, macrolides and azalides. The pathophysiological mechanism on which the Jarisch-Herxheimer reaction is based is not unanimous in the literature, and several theories are described such as an inflammatory reaction resulting from the phagocytosis of the microorganisms involved, endotoxemia and reaction to proteins and lipopolysaccharides resulting from bacterial death, which is the most accepted.^(61)^ The Jarisch-Herxheimer reaction in syphilis is more frequent in the exanthematous phase of recent syphilis, after the first dose of antibiotic. It is characterized by exacerbation of the existing syphilitic lesions on the skin, fever, chills, headache, nausea and arthralgias. Note that its occurrence does not justify interruption of the therapeutic regimen.^(29)^ In the management of pregnant women with the Jarisch-Herxheimer reaction, the use of common analgesics and antipyretics achieves spontaneous resolution of the condition within 12 to 48 hours. Sometimes it may be necessary to use corticosteroids and non-steroidal anti-inflammatory substances, depending on the severity of the dermal and arthralgic condition. Even though it is not a hypersensitivity (allergic) reaction to penicillin, it is extremely important to differentiate it from other pharmacodermias. Every pregnant woman with syphilis should be alerted to this possible reaction when penicillin therapy is instituted.^(1,39)^ Pregnant women should also be alerted about the possibility of triggering preterm labor.^(62)^

### How should syphilis treatment be controlled in pregnant women?

Even though the treatment of syphilis in pregnant women has known and widely disseminated guidelines, treatment control is one of its most critical points.^(63)^ As NTTs are titratable tests (VDRL, RPR or TRUST), they are used in this control. Control is performed monthly, preferably always using the same test, which allows a more reliable comparison between results. Quantification of the NTT titer must be obtained at the beginning of treatment (ideally, before or on the first day of treatment).^(39)^ Traditionally, treatment success is considered to be the reduction of the NTT titer by two dilutions (for example, 1/64 to 1/16) up to three months after the last dose of penicillin. Then, a reduction of three dilutions (for example, 1/64 to 1/8) for up to six months with progression until seroreversion (non-reactive NTT). The persistence of reactive results with low titers (1/1 to 1/4) for one year after treatment may indicate treatment success if new exposure during the analyzed period is ruled out (scar or serological memory).^(31,64)^ The possibility of reinfection or inadequate response to treatment must be considered when a previously treated pregnant woman meets one of the three criteria indicating the need for retreatment: 1) does not present the expected reduction in titers assessed by quantitative NTT; 2) shows an increase in titer by two or more dilutions in six months compared to the results of previous tests; or 3) shows recurrence of clinical signs and symptoms of syphilis. In these cases, a new treatment must be instituted as if it were late syphilis (three doses of benzathine penicillin 2,400,000 IU intramuscularly weekly for three weeks), extended to sexual partners. In these cases, investigation of neurosyphilis using cerebrospinal fluid puncture is recommended. If the cerebrospinal fluid test is compatible with neurosyphilis, treatment should be given with intravenous crystalline penicillin, as per the information in the part referring to the treatment of tertiary syphilis.^(29)^

### How to manage the partner of a pregnant woman with syphilis?

One of the chapters that generates more doubts in the approach to pregnant women with syphilis is the pharmacological conduct with the partner, who must also be treated. If he presents clinical manifestations of recent syphilis or presumed infection time of less than one year, a single dose of benzathine penicillin 2,400,000 IU intramuscularly is recommended. If he is asymptomatic and has reactive serology, he will be considered to have late latent syphilis, and the recommended dose is 2,400,000 IU of benzathine penicillin intramuscularly weekly for three weeks. However, if he is asymptomatic and has non-reactive serology, he should receive a single dose of benzathine penicillin 2,400,000 IU intramuscularly, considering the possibility of him being in a immunological window of opportunity.^(39)^ It is important to highlight that although the treatment of the sexual partner was removed from the criteria defining adequate maternal treatment at the end of 2017, this does not exempt him from being treated.^(65)^

## Gestational prognosis

Considering that the absolute majority of cases of syphilis in pregnant women are diagnosed in the recent and latent stages of the disease, compromising the embryonic and perinatal prognosis is normally the main concern. Adverse results translate into a higher frequency of miscarriages, prematurity, intrauterine growth restriction, low birth weight, fetal death and congenital syphilis. According to information in the literature, fetal loss (early and late) rates for these pregnant women are around 40%-45%.^(17)^ Preterm birth, low birth weight and clinical signs of congenital syphilis account for approximately 40% of cases.^(36-38)^ Due to such a challenging perinatal prognosis, screening and treatment of syphilis in pregnant women are recommended as early as possible.^(66)^ The possibility of congenital syphilis not manifesting at birth has made screening for maternal infection at birth a protocol conduct in peripartum care in Brazil.^(39)^ If the diagnosis of the mother is confirmed, the newborn is considered to have the disease and treated according to specific protocols for congenital syphilis. In mothers with a previous diagnosis, but without treatment or considered inadequately treated (inadequate regimen of benzathine penicillin for the syphilis phase, first dose of penicillin administered less than 30 days after birth, newborn with clinical or laboratory manifestations of syphilis), treatment and clinical follow-up of the newborn are also indicated.^(63)^

### Are there advantages of including the partner in antenatal care to control congenital syphilis?

Including the partner more actively in antenatal care will facilitate the prophylaxis, diagnosis and early treatment of syphilis in pregnant women, consequently avoiding congenital syphilis.^(67)^ In addition to this positive aspect of ultimately avoiding congenital syphilis, other benefits arise from antenatal care of the partner, such as the diagnosis of other diseases (hepatitis B and C serology, syphilis and HIV, high blood pressure, hyperlipidemia and diabetes) and the possibility of early treatments.^(68,69)^ In addition to offering the partner tests for diagnosis and treatment conditions for the aforementioned diseases, there is a reduction in domestic violence and an increase in natural breastfeeding rates.

### What would be the causes of failures in the control and prevention of vertical transmission of syphilis and possible ways to remedy them?

Despite the efforts in this direction, the development of vaccines against *Treponema pallidum* unfortunately is not yet a healthcare reality.^(70,71)^ Without this resource, antenatal care professionals need continuous attention and effort in the application of already known resources, including behavioral measures, always in line with care interventions. Constant and routine educational actions in sexual and reproductive health from the family, school, medical services and media in general are historically lost opportunities. It would be important to provide guidance on syphilis at all these levels, as well as on strategies to avoid becoming infected, with an emphasis on the regular and consistent use of male and female condoms.^(39)^ Considering the care aspects and since to date, vertical transmission rates of *Treponema pallidum* do not reflect the effort of interventions implemented with the aim to control it, it is imperative to seek more effective control measures to change this unfavorable course. If all protocols and guidelines published in Brazil were followed, congenital syphilis would already be under control. Therefore, what is missing is a central surveillance that regulates compliance with current protocols, creating non-punitive committees with power to promote reflection across the entire network involved in assistance in order to prevent cases of vertical transmission of *Treponema pallidum*.

Another factor with a negative impact on the success of syphilis treatment in pregnant women is the fear of an anaphylactic reaction with the use of benzathine penicillin, although the guidelines and clarifications issued by the Federal Nursing Council^(49)^ and the Ministry of Health clarify about its rarity.^(50)^

## Final considerations

Always consider that antenatal syphilis screening, its treatment and control are non-negotiable. To this end, the Ministry of Health recommends carrying out three diagnostic tests, depending on availability and local protocols, which may be serum tests (TT or NTT) or even a rapid test. Two tests will be carried out during antenatal care (at the first consultation and in the transition between the second and third trimesters of pregnancy) and the third at the time of delivery.^(39)^ Also remember to search for syphilis infection also after cases of miscarriage and ectopic pregnancy. Whenever a prenatal syphilis diagnosis test is requested, the return should be as fast as possible, so that the pregnant woman can be treated quickly. In general, good laboratories already perform the confirmatory test on the same sample if the first test was reactive. In difficult-to-access regions, it is legal to treat pregnant women based only on the screening exam. Although far from the correct and ideal situation, this is the measure with the least harm to families. It is important to remember that screening does not end during antenatal care, but rather in the peripartum period. Infections acquired late and not reported may be identified at the time of birth, and the follow-up of these children will depend on the action of pediatric professionals, ensuring that cases of congenital syphilis are not missed, especially those that present late. The absolute indication of a single dose of benzathine penicillin 2,400,000 IU intramuscularly for the treatment of syphilis in pregnant women in recent syphilis remains unquestioned. For late syphilis, the prescription is 2,400,000 IU intramuscularly, weekly, for three weeks. Remember to treat the partner. For penicillin allergy with a risk of anaphylaxis, it is prudent to correctly perform the diagnosis and desensitization in a hospital setting, preferably by allergists.

In the case of sexual violence against a pregnant woman by an unknown aggressor without the possibility of carrying out laboratory tests, a single dose of benzathine penicillin G 2,400,000 IU intramuscularly must be administered prophylactically. In summary, one of the main principles for the successful control of congenital syphilis is not to miss any opportunity at any time or in any clinical situation.^(1,72,73)^

Another measure that could objectively reduce new cases of maternal syphilis (prophylaxis) and help in the identification and treatment of evolving cases would be including the partner in antenatal care, thereby offering in exchange the probability of a healthy fatherhood, mother without the diagnosis of syphilis and son free from the spectrum of congenital syphilis. Considering this is essentially a sexually transmitted infection, it is logical to assume that control will not be possible with unilateral measures.^(67,68)^
